# Combined ultrasound-ozone treatment for reutilization of primary effluent—a preliminary study

**DOI:** 10.1007/s11356-020-10467-y

**Published:** 2020-08-20

**Authors:** Giada Rossi, Matia Mainardis, Eleonora Aneggi, Linda K. Weavers, Daniele Goi

**Affiliations:** 1 Polytechnic Department of Engineering and Architecture (DPIA), University of Udine, Via del Cotonificio 108, 33100 Udine, Italy; 2 Ohio Water Resources Center, Department of Civil, Environmental, and Geodetic Engineering, The Ohio State University, 470 Hitchcock Hall, 2070 Neil Ave., Columbus, OH 43210 USA

**Keywords:** Water recovery, Ozonation, Ultrasound, Disinfection, Primary effluent, AOP

## Abstract

The present work is a preliminary study on the potential of low-frequency ultrasound irradiation coupled with O_3_ process for the disinfection of a primary effluent from a municipal wastewater treatment plant preserving nutrient levels (in particular nitrogen and phosphorous), for its possible reuse in civil, industrial, and agricultural sectors. The treated water could be reused, after appropriate dilution, contributing to the circular economy perspective and reducing the need for both chemical fertilizer addition and freshwater supply. The effect of different specific ultrasonic energies and ozone doses was assessed on a bench-top system, composed of an ultrasonic reactor and a semi-batch ozonation vessel. The results showed that the combined US-O_3_ process produces a good removal efficiency regarding soluble Chemical Oxygen Demand, sCOD (ca. 60%), anionic surfactants (ca. 50%), and formaldehyde (ca. 50%), and an optimal abatement for Methylene Blue Active Substances (MBAS, > 90%). The process also reached high disinfection performances, obtaining 4 logs for *E. coli* and 5 log abatement for Total Coliforms. The high removal efficiency is matched by an outstanding retention of nutrients (total nitrogen and orthophosphate) highlighting a high potential value for agricultural reuse of the treated primary effluent, with possible significant saving of chemical fertilizers. It was concluded that low-frequency ultrasound pre-treatment, combined with ozonation, could be a useful process for primary effluent recovery for several purposes. Further studies are expected to be planned and executed to evaluate system scale-up feasibility.

## Introduction

Fresh water scarcity has become one of the most significant environmental challenges in the twenty-first century, mainly caused by an uneven distribution of water resources throughout the world (Jasim et al. 2016). The stress on water sources is expected to further increase in the next years, due to population growth, surface water and groundwater pollution, droughts, and climate changes (Farhadkhani et al. 2018).

The agricultural sector accounts for about 70% of total water usage (Jasim et al. 2016). Nowadays, it appears unavoidable to boost for treated wastewater reuse in this sector, as an essential component of an integrated and sustainable water resources management (Farhadkhani et al. 2018). In response to the increasing agricultural demand for water, coupled with water shortages, countries such as Israel, Singapore, and Australia recently adopted water reclamation programs (Savchenko et al. 2019). However, reclaimed wastewater reuse for irrigation purposes may create risks to human and environmental health, due to the toxic substances that can remain in the treated wastewater (Li et al. 2019). Reclaimed water contains valuable components, in particular nutrients (nitrogen and phosphorous), potentially reducing the need for nutrient addition to the agricultural lands, with a global positive environmental and economic impact.

Primary effluents (prEFFs) from wastewater treatment plants (WWTPs) have a good agricultural reuse potential, because most of the nutrients are conserved throughout the primary treatment, while only high-density particulate matter is removed (Abdessemed and Nezzal 2003). However, a careful monitoring of residual microorganism contamination should be performed to prevent infectious parasites diffusion in agricultural crops (Leonel et al. 2016). At the present, treated municipal wastewater reuse is limited worldwide (< 2.5%) and should be boosted in particular in dry areas, with a strong focus on public concern regarding human and environmental health (Deviller et al. 2020). The most important aspect for wastewater recovery from WWTPs is disinfection: as a first target for reuse, water must be safe from a microbiological point of view. Advanced oxidation processes (AOPs), including Fenton or Fenton-like, ozonation, sonolysis, and photocatalysis, have been widely investigated to abate bio-recalcitrant pollutants in wastewater streams (Chakma and Moholkar 2016). Ozone, in particular, has a strong potential for both disinfection over a broad range of applications (Lazarova et al. 2013) and partial mineralization of electrophilic pollutants. Usually, ozone treatment is used to improve the performances of a subsequent biological process, where the nutrient charge is dramatically reduced, or to abate recalcitrant COD after secondary biological process (Mainardis et al. 2020). In recent years, more advanced ozonation applications gained attention, as an economical option for advanced wastewater treatment, due to a significant capacity for emerging contaminants oxidation and disinfectant capability (Nasuhoglu et al. 2018; Yang et al. 2019). Ozone disinfection of wastewater from primary treatment could reduce the pathogenic risk and the microbial toxicity of the effluents, degrading electrophilic organics and maintaining, at the same time, nitrogen and phosphorus. Consistently, a high disinfection efficiency of ozone in removing enteric pathogens (such as *E. coli*) was reported in Gomes et al. (2018).

Low-frequency ultrasound (US) is another AOP, widely investigated in water treatment technologies (Mahamuni and Adewuyi 2010), even if full-scale application has proved to be cumbersome due to the high energy expenses (Al-Juboori et al. 2016). US waves with enough pressure amplitude generate cavitation bubbles (Shah et al. 2012) that collapse resulting in extreme localized temperature and pressure in solution (up to 5000 °C and 500 atm) (Ohrdes et al. 2018) and free radical formation, able to attack the biological contaminants (Pokhrel et al. 2016). Low-frequency ultrasounds are particularly indicated in wastewater treatment, positively affecting US activity (Al-Bsoul et al. 2020) and producing larger cavitation bubbles that result in higher energy release (Al-Bsoul et al. 2010; Young 1989).

Apart from separated AOPs, hybrid solutions, which couple two or more different processes, are particularly indicated in degrading recalcitrant pollutants (Chakma and Moholkar 2013). Ozone, as an example, was shown to be more efficient than other AOPs when coupled with electrocoagulation in greywater treatment (Barzegar et al. 2019). The enhanced efficiency of combined US, O_3_, and other oxidation treatments for wastewater oxidation and disinfection was proved in literature studies. The potential benefit of a combined sonozone treatment relates to sonication-induced pressure waves that, besides stimulating the production of hydroxyl radicals, might augment ozone transfer rate into water (Yargeau and Danylo 2015). Consistently, an augmented presence of hydroxyl radicals was reported in a combined US/ozone process in sulfamethoxazole treatment in the work by Guo et al. (2015), with a significant increase in the reaction rate under different pH conditions. Weavers et al. (1998) studied sonolysis, ozonolysis, and a combined process to degrade three known organic pollutants, while Wang et al. (2012) combined sonication and ozonation to remove tetracycline, and Yargeau and Danylo (2015) applied these AOPs to ibuprofen oxidation in water. The combined ultrasound and ozonation process has proved to be more efficient than ultrasound and ozonation alone in the treatment of real abattoir wastewater, showing significant biochemical oxygen demand (BOD) and COD removal, coupled with a good effluent disinfection, due to the complete inactivation of Total Coliforms (Alfonso-Muniozguren et al. 2020). A combined sonozone treatment of natural organic matter was proposed in Olson and Barbier (1994), proving an enhanced Total Organic Carbon (TOC) removal, if compared with ozone treatment alone. However, limited experiences were reported on combined US and O_3_ application on primary wastewater effluent treatment.

The advantages of a combined US-O_3_ process, according to relevant literature studies, are briefly summarized in Table 1.

**Table 1:** Advantages of combined sonication and ozone process

Advantage	References
Sonication-induced pressure waves stimulate hydroxyl radicals formation	Guo et al. (2015), Yargeau and Danylo (2015), Wang et al. (2012)
Sonication-induced pressure waves augment ozone transfer rate	Yargeau and Danylo (2015), Wang et al. (2012)
Increase process efficiency (COD and BOD removal) if compared with separate treatment	Alfonso-Muniozguren et al. (2020), Wang et al. (2012)
Good effluent disinfection	Alfonso-Muniozguren et al. (2020)
Enhanced total organic carbon (TOC) removal	Weavers et al. (1998), Olson and Barbier (1994)
Combined process more efficient at low frequency (20 kHz)	Weavers et al. (1998)

In this work, a combined US-O_3_ treatment of a prEFF coming from a municipal WWTP was studied under different operating conditions, to evaluate the system efficiency. The experiments were aimed at determining if sonication followed by ozonation is able to improve prEFF characteristics, while preserving nutrients (in particular nitrogen and phosphorous), allowing for a potential and safe effluent reuse. The main target of the experimental tests was to recover the primary effluent for several purposes (civil, industrial, and agricultural), with a special focus on agricultural irrigation. Therefore, some meaningful characterization parameters (in particular the ones exceeding the acceptable limits for Italian legislation), were controlled throughout the tests. The recovery of water and nutrients contributes to the circular economy perspective through the reduced need for both chemical fertilizers and freshwater supply in the agricultural sector. This approach allows to transform WWTPs in sustainable technological systems (Leyva-Diaz et al. 2020; Qu et al. 2019).

## Materials and methods

### Primary effluent characteristics

The prEFF used in the experimental tests was collected from the primary settling tank of a municipal WWTP, having potentiality of 200,000 population equivalent, located near Udine (northeast of Italy). The average influent flowrate of the plant was 350 L/s. The samples were collected in grab mode two times per week and were stored overnight in a refrigerator before executing the tests. The primary clarification and sedimentation in the analyzed WWTP were performed with the addition of coagulants (alum and ferrous sulfate), allowing heavier particles to be efficiently removed.

### Bench-top ultrasonic and ozonation apparatus

The bench-top treatment consisted of two stages: the first stage was a continuous mode ultrasonic reactor, in which prEFF flowed through the device at various retention times. In the second stage, the US pre-treated water was ozonated in semi-batch mode at established O_3_ doses and contact times.

**Fig. 1:**
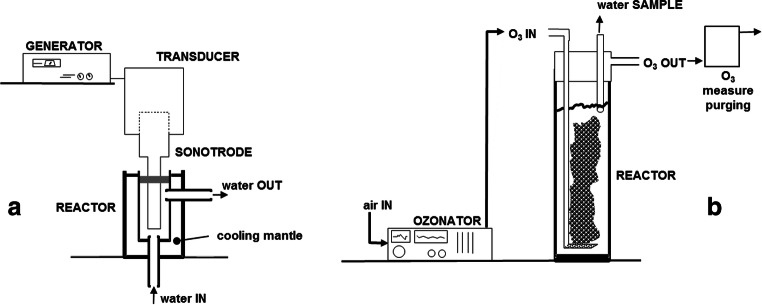
Bench-top equipment used for the experimental tests. **a** Ultrasonic reactor operating in continuous flow mode. **b** Semi-batch ozone reactor, with prEFF in batch mode and ozone gas in continuous mode

The US equipment (Fig. 1a) was a Dr. Hielscher GmbH (UIP 250 model), characterized by 250 W power, with a 24 kHz ultrasonic transducer having a BS24 titanium probe (22-mm diameter), operating at 20% amplitude. A similar apparatus was used for activated sludge US treatment in Simonetti et al. (Simonetti et al. 2014) and was applied to cheese whey pre-treatment before anaerobic digestion process in Mainardis et al. (Mainardis et al. 2019). The probe was placed in a D22K flow vessel. The reactor had a diameter (*D*) of 35 mm, a height (*H*) of 50 mm, and an internal volume (*V*) of 48 mL. When the titanium probe was immerged into the reactor, the available fluid volume was 38 mL, with maximum optimization of cavitation performance by simple horn.

The wastewater flowed into the reactor by means of a peristaltic pump (Cellai Perinox SF3, 230 V, 80 W, 1.6x1.6 3R) with flowrate ranging from 7 to 23 L/h. The selected ultrasonic contact times were 6, 12, and 20 s, corresponding respectively to 10 (US-1), 20 (US-2), and 50 (US-3) kJ US energy/L. US power was verified by calorimetric measurements (Wei et al. 2015), and the sonochemical efficiency in the whole reaction system was quantified using the Weissler reaction (Kimura et al. 1996). The reactor was equipped with a water-cooling mantel, to keep the temperature at 20 °C during the tests. This temperature value was selected to be consistent with the mean wastewater temperature in the selected WWTP.

The power of ultrasound device has been chosen by evaluating recent literature outcomes that showed excellent results through the use of low-frequency (20–40 kHz) ultrasound with medium-low power (Naddeo et al. 2014). Moreover, low-power ultrasound has been chosen to reduce the total energy consumption levels.

US calibration was performed using standardized KI solution (0.1 M), as proposed in Koda et al. (2003). The increase in UV absorbance is linked to I^−^ reaction with H_2_O_2_ (produced by ultrasound in water) with formation of I_2_. In the calibration phase, a linear correlation between the sonication time (6–100 s) and the measured UV absorbance was observed for different amplitudes (20%, 50%, 100%) of the US device, at a fixed wavelength of 355 nm. A similar linear correlation emerged between sonication time and absorbed US energy (Wh). An amplitude of 20% was selected for successive tests, given the highest linearity (*R*^2^ = 0.995) among the various tested amplitude values, together with the reduced energy need (80 W). The sonotrode BS24 was selected as the one which showed the best performances among the available sonotrodes. The ultrasound power in the different operating conditions was measured using an energy consumption meter.

The ozonation system (Fig. 1b) was assembled in semi-batch mode, continuous with respect to gas-ozone flow and batch with respect to liquid. The 60-mm diameter glass reactor was 400-mm tall (1.5 L volume) and contained a sintered-glass fine porous-bottom diffuser (porosity 2) to introduce ozone in the reactor. Two washing bottles in series, containing 2 M KI solution (Tjahjanto et al. 2012), trapped residual gaseous O_3_ at the outlet of the reactor. All ozonation tests were conducted introducing 1 L of sonicated prEFF in the ozone reactor under constant room temperature (20 ± 1 °C) conditions.

A 180 W, C-Lasky CL-010-DT model (AirTree Ozone Technology Co.) O_3_ generator, with a maximum ozone generating capacity of 2 g/h (10 L/min of air flow) was used. A 3 L/min air flowrate was maintained and monitored during the experiments using an internal flow meter, resulting in an ozone production rate of 8.00 mgO_3_/min during the tests. Ozone gas production and flowrates were calibrated and monitored with high reproducibility using the iodometric method (method 2350 E of Standard Methods for the Examination of Water and Wastewater) (APHA 2012).

Three different ozonation times (150, 300, 650 s) were tested to reach three different ozone concentrations in the liquid mixture, respectively 20, 40, and 87 mg of ozone supplied to the reactor. The O_3_ doses transferred into the solution were calculated by influent and effluent gas-phase ozone measurements. Ozonated air flow was supplied to the reactor for the desired pre-set times. Once the planned ozone dose was reached, ozonation was stopped, and the reaction was allowed to continue for 10 minutes, prior to samples withdrawal for analysis.

### Chemical and microbiological analysis

Meaningful chemical and microbiological parameters were monitored basing on prEFF characteristics, water reuse potential, and regulation targets. Soluble Chemical Oxygen Demand (sCOD) and UV254 were measured as surrogate parameters of the organic carbon content and the organic micropollutants that can be leached by the treatment (Bahr et al. 2007). Total nitrogen (TN) and orthophosphate represented nutrient concentration. Methylene blue anionic surfactants (MBAS) were analyzed as a common measure for anionic surfactants present in the sewage. Formaldehyde was monitored as well, since it is typically highly present in prEFF and is also recognized as an ozonation byproduct (Wert et al. 2007). Total Coliforms and *E. coli* were used as indicator parameters of microbiological water quality.

Before chemical parameter examination, each sample was filtered through a 0.45-μm Whatman® glass microfiber filters, Grade GF/A, to prevent residual particulate interferences during the successive measurements. TN was measured using a TOC/TN-TOCN-4110 Analyzer (Shimadzu). UV254 was determined with a Hach-Lange DR5000 spectrophotometer, and formaldehyde was detected using MBTH method HACH kit test. All the other chemical parameters were measured following Standard Methods (APHA 2012); in particular, sCOD was evaluated using method 5220D, orthophosphate using method 4500-P C, and MBAS using method 5540 C. Microbiological characterization was performed using membrane filtration method 9222, utilizing ECD MUG agar (Fluka) for the Total Coliforms and *E. coli*. All the analytical measurements were performed in triplicate, with standard deviation ranging from 5 to 15%; in the following, the mean values were reported for each analyzed parameter.

### Simplified energy analysis

The specific energy consumption of US and ozone apparatus was calculated for the different operating conditions from the instrument powers, the results of the energy meter measurements and the treated wastewater volume (see the “Bench-top ultrasonic and ozonation apparatus” section). Mean Italian electricity cost of 0.20 €/kWh (ARERA 2019) was considered for the simplified energy analysis.

## Results and discussion

### Wastewater characterization

The prEFF exhibited an average abatement of 20–25% of tCOD and 50–60% of TSS if compared with the raw influent wastewater. The average prEFF characteristics are reported in Table 2. It could be noted that the primary effluent was characterized by a moderate organic charge (tCOD of 225 mg/L and sCOD of 117 g/L) and a good nutrient concentration (TN up to 21.13 mg N/L and orthophosphate concentration of 5.80 mg P/L), while suspended solid material and turbidity were comparable with other WWTPs in the analyzed area. In Table 2, the limits for irrigation, as specified in the current Italian Regulation (DM 185/2003), were reported as well. The characteristics of the analyzed prEFF were generally suitable for agricultural irrigation water reuse, if the critical parameters (in particular microorganism concentration) could be contained under acceptable values. In the past, in the selected WWTP, a partial water reuse was planned, considering a mixture of prEFF with the final WWTP effluent to amend and irrigate yields and crops in the surrounding agricultural area. The actual wastewater characteristics, in terms of microorganism concentration, were comparable with what was reported in Campos et al. (2016), where mean *E. coli* concentration from 4 different primary effluents was claimed to be 3.06 × 10^6^ CFU/100 mL. In the work by AlMomani and Ormeci (AlMomani and Ormeci 2016), instead, a generally more concentrated primary effluent was reported, having a sCOD concentration of 242.0 mg/L, TN of 41.0 mg N/L, and TP up to 10.0 mg P/L, before application of microalgae treatment.

**Table 2:** Primary effluent characterization and acceptable limit for irrigation reuse

Parameter	Units	Value	Limits for irrigation*
pH	-	7.8	6–9.5
TSS	mg/L	75	10
Turbidity	NTU	45	-
tCOD	mg O_2_/L	225	100
sCOD	mg O_2_/L	117	-
DOC	mg C/L	121	-
UV254nm	cm^−1^	2.48	-
TN	mg N/L	21.13	15
PO_4_^3-^	mg P/L	5.80	2
MBAS	mg MBAS/L	1.25	0.5
Formaldehyde	mg H_2_CO/L	1.03	0.5
Total Coliforms	CFU/100 mL	1.0 × 10^7^	-
*E. Coli*	CFU/100 mL	7.0 × 10^5^	100

*Acceptable limits from Italian Regulation DM 185/2003 (Italian Government 2003)

It should be pointed out that the legislation limits for nutrients, reported in Table 2, can be increased to 35 mg N/L (as for nitrogen) and 10 mg P/L (as for phosphorous), if the considered receiving soil does not fall in the “nitrate-vulnerable” areas. In addition, the applicable regulation for agricultural reutilization significantly varies from country to country, so the effective choice of the technology to apply prior to reuse should consider local wastewater characteristics and legislation limits, to define the correct process approach to agricultural irrigation planning and design.

### Ozone consumption for the different operating conditions

**Fig. 2:**
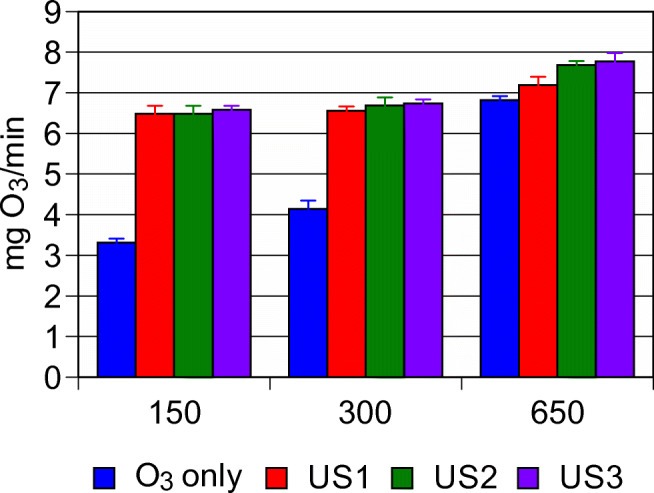
Ozone demand in various reaction conditions (process scheme: O_3_, ozonation alone; US-1 + O_3_, 6 s of ultrasonic contact time + ozonation treatment; US-2 + O_3_, 12 s of ultrasonic contact time + ozonation treatment; US-3 + O_3_, 20 s of ultrasonic contact time + ozonation treatment)

As a first step for evaluating combined US-O_3_ treatment performances, a measure of the ozone consumed during the whole semi-batch treatment was carried out. Three different ozonation times (150, 300, 650 s) and four process schemes (O_3_ alone, US-1 + O_3_, US-2 + O_3_, US-3 + O_3_) were tested. Residual ozone was measured at the end of the reaction, and the results are depicted in Fig. 2. It could be noted that US pre-treatment led to a general increase in ozone demands. An augment of ultrasonic contact time slightly affected the ozone demand, even though a general small increase could be observed. The maximum oxidation potential of the system has been obtained at the maximum ozonation time (650 s) and the highest-energy US-3 pre-treatment.

### Effect of ultrasonic pre-treatment on organic material ozonation

**Fig. 3:**
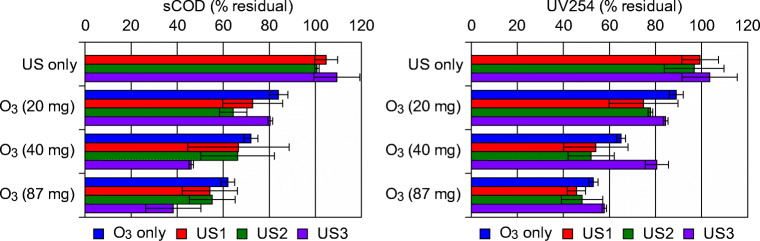
sCOD and UV254 removal during the treatment tests. (Reaction conditions: O_3_, ozonation alone; US-1 + O_3_, 6 s of ultrasonic contact time + ozonation treatment; US-2 + O_3_, 12 s of ultrasonic contact time + ozonation treatment; US-3 + O_3_, 20 s of ultrasonic contact time + ozonation treatment. Ozonation times of 150, 300, 650 s have been used corresponding, respectively to 20, 40 and 87 mg ozone concentration)

In Fig. 3, the results of the tests regarding sCOD and UV254 parameters are depicted. When ultrasound treatment alone was applied to the prEFF, a measurable sCOD increase was observed, even at short sonication times. This outcome could be explained with the physicochemical modification of the mixture during the sonication process that augmented the fraction of soluble organic material. UV254 parameter, similarly, showed a moderate increase during the sole sonication process.

The sole ozonation led to a maximum 40% sCOD removal, while the combined US-O_3_ treatment allowed a general increase of sCOD abatement. sCOD reduction was almost linear with US dose, reaching a maximum of 60%, when the high-intensity US-3 pre-treatment (50 kJ US energy/L) was performed. The high removal capacity of sCOD in the US-O_3_ combined system is in agreement with the study by Guo et al. (2015) related to sulfamethoxazole degradation. They found an increase of the process kinetics (6–26%) correlated to the higher radical number generated by the ultrasound/ozone treatment. The organic material solubilized in the liquid medium by providing additional ultrasonic energy seemed to be more oxidizable and mineralizable in the subsequent ozonation process.

UV254 parameter showed a similar trend as sCOD, when O_3_ and US treatments were applied individually. However, in the combined process, a higher removal was obtained for lower US energy (for US3-O_3_, a reduction higher than 50% was achieved). A detrimental effect was observed when increasing ultrasound energy, probably due to a release of higher amounts of UV-absorbing refractory compounds in the high-energy US process. The results for sCOD and UV254 removal (around 60% and 40%, respectively) compared with sole ozonation are consistent with the data reported for a primary effluent by Marce et al. (2016), where a reduction of 60% for COD and 40–45% for UV254 was observed.

### Ultrasonic pre-treatment effect on nutrient ozonation

**Fig. 4:**
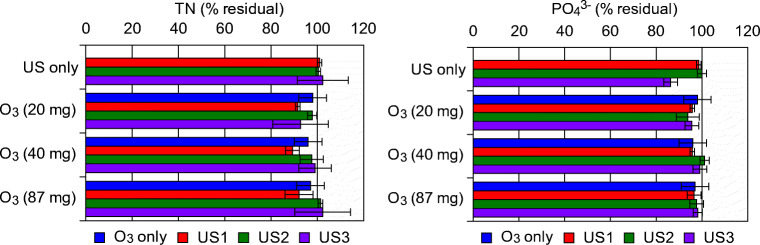
TN and PO_4_^3−^ removal during the combined US-O_3_ treatment tests (reaction conditions: O_3_, ozonation alone; US-1 + O_3_, 6 s of ultrasonic contact time + ozonation treatment; US-2 + O_3_, 12 s of ultrasonic contact time + ozonation treatment; US-3 + O_3_, 20 s of ultrasonic contact time + ozonation treatment. Ozonation times of 150, 300, and 650 s have been used corresponding, respectively to 20, 40, and 87 mg ozone concentration)

At the selected US amplitude of 20%, the electric power consumed by the US device was measured as 80 W. In Fig. 4, the trend of TN and orthophosphate concentration throughout the tests is depicted. Considering ultrasonic process alone (without ozonation), TN remained rather constant. At the highest applied US energy, a minor TN increase was observed, probably bound to some release of nitrogenous material from the particulate matter. This outcome is coherent with what reported in Garoma and Pappaterra (Garoma and Pappaterra 2018) that claimed a moderate increase of about 6% in the soluble nitrogenous fraction, when applying US pre-treatment to digestate, before anaerobic digestion. Ozonation treatment alone, instead, had no measurable effect on TN.

The combined US-O_3_ process led to a moderate nitrogen abatement of about 10%, when a low ultrasonic energy dose was used. An increase in TN was observed with augmenting US energy that could be explained with nitrogen compounds that moved from the particulate to the soluble fraction after sonication treatment. The initial C/N ratio of 5.7 decreased to 3.1–3.8 due to the abatement of 40–60% of the organic material during the combined US-O_3_ process, indicating a high potential value for agricultural reuse of the treated primary effluent, with possible significant saving of chemical fertilizers, given the good preserved nutrient concentration.

Orthophosphate concentration did not substantially change throughout the tests, both when ultrasound and ozonation were applied alone and in the combined US-O_3_ prEFF treatment, again underlining a favorable condition for agricultural reuse of the treated effluent. Ultrasound and ozonation were studied both singularly and in combination to recover microalgal bio-compounds and phosphorous in the work by Gonzalez-Balderas et al. (2020), highlighting protein and lipid release in the US process, while ozone stimulated carbohydrate and phosphorous unbinding (Gonzalez-Balderas et al. 2020). Similarly to what was obtained in the present study, the combined process achieved high recovery yields, showing a good potential for valuable compound production.

### Effect of ultrasonic pre-treatment on MBAS and formaldehyde ozonation

**Fig. 5:**
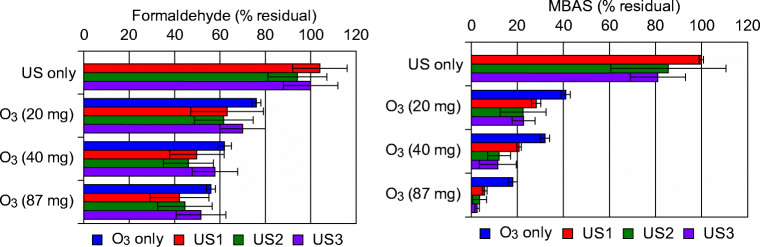
Formaldehyde and MBAS removal during the treatment tests (reaction conditions: O_3_, ozonation alone; US-1 + O_3_, 6 s of ultrasonic contact time + ozonation treatment; US-2 + O_3_, 12 s of ultrasonic contact time + ozonation treatment; US-3 + O_3_, 20 s of ultrasonic contact time + ozonation treatment. Ozonation times of 150, 300, and 650 s have been used corresponding, respectively to 20, 40, and 87 mg ozone concentration)

MBAS and formaldehyde removal results are summarized in Fig. 5. Formaldehyde concentration in the aqueous solution remained substantially stable after US application alone, even if a minor releasing effect was observed (in particular in US-1 test).

Ozonation is well-known to produce formaldehyde (and aldehydes in general) as an oxidation byproduct of water and wastewater that pose potential health risks (Papageorgiou et al. 2017). In the present experimental tests, however, ozonation treatment led to a slight removal of the high formaldehyde concentration originally present in the prEFF. This removal was enhanced in the combined US and O_3_ treatment, where the reduction reached about 40% and 60% respectively in US-1 and US-2 tests, while the US-3 treatment resulted less effective (removal in the range 30–50%). MBAS concentration showed a meaningful behavior during the tests. When US treatment was applied alone, MBAS abatement reached low levels (maximum of about 20%) in the US-3 treatment, while ozonation alone led to a medium to high removal, in the range of 60–80%. The ozonation process preceded by US showed a dependence over the O_3_ concentration, with an excellent removal when higher doses (40 and 87 mg O_3_) were applied. In particular, when the maximum ultrasonic energy (US-3) was tested, MBAS reduction was over 95%. The good MBAS reduction obtained in the combined treatment could be ascribed to the reduction of surfactant chain length stimulated by US pre-treatment, and the subsequent optimal mineralization in the ozonation process. However, this assumption needs to be further investigated in successive studies. The beneficial effect of O_3_ treatment in MBAS removal is well-known in literature; as an example, in the work by Turkay et al. (2017), ozonation improved MBAS removal of the combined electro-peroxone process from 77 to 86%.

### Ultrasonic pre-treatment effect on ozonation disinfection

The disinfection performances of the combined US-O_3_ treatment on the prEFF were measured by means of the *E. coli* and Total Coliform parameters.

**Fig. 6:**
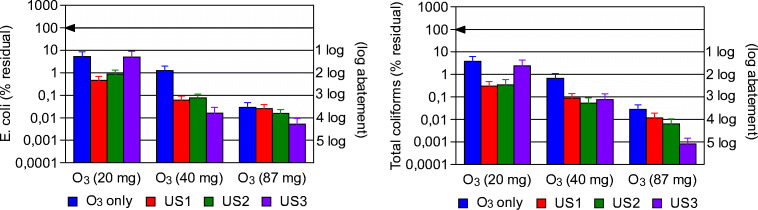
Disinfection efficiency of the US-O_3_ combined treatment (reaction conditions: O_3_, ozonation alone; US-1 + O_3_, 6 s of ultrasonic contact time + ozonation treatment; US-2 + O_3_, 12 s of ultrasonic contact time + ozonation treatment; US-3 + O_3_, 20 s of ultrasonic contact time + ozonation treatment. Ozonation times of 150, 300, and 650 s have been used corresponding, respectively to 20, 40, and 87 mg ozone concentration)

A negligible disinfection was observed when the sole US treatment was applied (data not shown), while when ozonation alone was performed, an evident microorganism inactivation, exceeding 3 logs for both Total Coliforms and *E. coli* (Fig. 6), appeared at the maximum applied dose (87 mg O_3_). Gomes et al. (2018) compared the effectiveness of biological filtration (using *C. Fluminea*), ozonation, and photocatalytic oxidation for *E. coli* removal from canal water. As for ozonation treatment, they observed a low removal for an applied O_3_ dose of 0.05 mg O_3_/L, while a complete abatement was obtained increasing ozone dosage to 0.16 mg O_3_/L (O_3_ dose was significantly lower than the concentration used in the present study). *E. coli* efficiency reported in Gomes et al. (2018) was higher than that highlighted in the present work, mainly due to the different wastewater characteristics: initial *E. coli* concentration in Gomes et al. 2018 was about 1.0 × 10^3^ CFU/100 mL, more than two order of magnitudes lower than the actual prEFF, as shown in Table 2).

Once sonication pre-treatment was introduced, ozonation efficiency appeared to increase, and for the applied ultrasonic energy US-3, a maximum disinfection rate around 5 log was obtained for Total Coliforms. This enhanced disinfection efficiency could be ascribed to the US-related removal of the microorganisms from the suspended particles wherein they could be hidden, increasing microorganism availability in the liquid medium and thus allowing ozone disinfection to be more effective. When the maximum sonication energy (US-3) and the minimum O_3_ dose (20 mg) were applied, a lower disinfection efficiency was observed compared with the higher ozone doses. This behavior could be related to sonication effect on suspended particles, with the release of significant amounts of scavenging material, that probably reduced ozone disinfection power. These basic tests can be useful to establish the O_3_ dose to apply to obtain the desired disinfection efficiency in the combined US-O_3_ system for agriculture wastewater reutilization.

Regarding ozone residual toxicity, Mainardis et al. (2020) showed that ozone as a tertiary treatment after conventional biological activated sludge did not substantially modify toxicity characteristics of the treated effluent, highlighting the feasibility to substitute full-scale physicochemical coagulation-flocculation process with ozonation. Moreover, recently, ozone was shown to efficiently degrade pharmaceuticals in wastewater, while a further polishing phase with suspended biofilm carriers was proposed to reduce effluent toxicity (Tang et al. 2019). Barzegar et al. (2019), instead, studied a combined electrocoagulation and ozone treatment for greywater reuse, having COD concentration of 460 mg/L (higher than the analyzed prEFF), Total Coliforms in the range of 2–5 × 10^7^ CFU/100 mL (comparable with that of actual wastewater) and *E. coli* concentration of 2–2.8 × 10^3^ CFU/100 mL (lower than actual prEFF). They reported a higher COD removal in the combined process (final COD of 53 mg/L), if compared with the actual obtained COD efficiency, while they registered a lower disinfection effect (4 logs as for Total Coliforms and 2 logs as for *E. coli*) if compared with the COD abatement and disinfection efficiency obtained in the present study.

Summarizing, the combined US-O_3_ process can efficiently remove chemical and biological contaminants form prEFF, preserving nutrient level. The comparison of literature results is tricky due to the many parameters that are taken into consideration and that influence the activity. However, it was found that the present results are in good agreement with previously reported data (depending on the analyzed parameters).

### Energy analysis

The results of the basic energy analysis are summarized in Table 3. It could be seen that the electricity consumption for US pre-treatment was in the range of 2.78–13.9 Wh/L, and was generally lower than ozonation energy expense (range of 7.5–32.5 Wh/L). The combined US-O_3_ energy cost was evaluated to be in the range of 2.16–9.28 €/L, considering the actual Italian energy market. This cost can be compared with freshwater supply cost in Italy, that is actually in the range of 0.79–1.62 €/L (ARERA 2019), and is generally cheaper than that observed in most European countries. In regions where water availability is scarce (and consequently freshwater price is high, for example, when seawater desalination is performed), the sonication-ozonation process appears economically sustainable and feasible, but should be supported by a pilot campaign to get more robust data. The application of the proposed technology in remote and decentralized areas could be boosted by integration of solar energy, providing a significant share of the needed electricity in a clean way.

**Table 3:** Evaluation of energy consumption and energy costs for applied O_3_ and UV treatments

Test	Treated wastewater (L)	Specific electricity consumption (Wh/L)	Specific electricity cost (€/L)
O_3_ (20 mg)	1	7.5	1.5
O_3_ (40 mg)	1	15	3
O_3_ (87 mg)	1	32.5	6.5
US-1	0.038	2.78	0.56
US-2	0.038	5.56	1.11
US-3	0.038	13.9	2.78

Considering the results of the experimental campaign of the combined US-O_3_ treatment (see the “Effect of ultrasonic pre-treatment on organic material ozonation,” “Ultrasonic pre-treatment effect on nutrient ozonation,” “Effect of ultrasonic pre-treatment on MBAS and formaldehyde ozonation,” and “Ultrasonic pre-treatment effect on ozonation disinfection” sections) and the Italian legislation limits for agricultural reutilization (Table 2), it could be stated that probably some dilution of the treated prEFF (for example with secondary WWTP effluent) would be needed to fit all the required parameters, in particular, regarding formaldehyde and microorganism concentration.

### Final considerations for reutilization perspective

The nutrient content of the investigated primary effluent was in line with the nutrient limits for agricultural irrigation, as imposed by Italian legislation for “non-nitrate-vulnerable” areas. The present effluent had nutrient concentrations of 21.13 mg N/L and 5.80 mg P/L against limit values of 35 mg N/L for total nitrogen and 10 mg P/L for phosphorous. These values were not appreciably altered by the combined US-O_3_ treatment. MBAS (80–90%) and Total Coliform (reduction to log 5) removal during US-O_3_ treatment was meaningful, lowering the initial concentrations to values generally suitable for irrigation purposes. After the applied sonication-ozonation treatment, tCOD (40–60% of abatement), formaldehyde (40–60% of reduction), and *E. coli* (reduction to log 4) were dramatically lowered, even if the obtained outcome was not sufficient to satisfy Italian legislation limits (some dilution would be needed). Considering the concentration variability in the primary effluent and Italian legislation limits, it could be assumed that, to be sure to respect the limits, a dilution factor of the treated prEFF should be necessary. The dilution could be done with the secondary WWTP effluent. Anyway, through the proposed system, a reduction of both chemical fertilizer addition and freshwater supply in the agricultural sector could be obtained, contributing to circular economy perspective. Summarizing, the ultrasound-ozone technology was shown to be suitable to allow the reuse of primary wastewater effluent, after proper dilution with, as an example, a secondary WWTP effluent.

Treated wastewater reutilization can reduce the huge chemical fertilizer consumption in the agricultural sector, in particular where intensive agriculture is applied. An interesting study (Sun et al. 2019) reported a mean consumption of chemical fertilizers in China up to 521.6 kg/ha, with total yearly consumed amount of 59.8 million tons fertilizer. Thus, the opportunity of valorizing the nutrients contained in wastewater should be deepened in further studies, to avoid an excessive chemicals’ usage in the soil, that can create serious adverse environmental externalities, such as diffused source pollution (Sun et al. 2012) and Greenhouse Gases (GHG) emissions (Zhu et al. 2006).

A successive experimental phase is needed to evaluate in detail the influence of the main operating parameters on the efficiency of the proposed combined US-O_3_ process, with a stronger focus on effluent toxicity characteristics. Measuring the bubble size in ozone treatment, in addition, would be beneficial to understand the process dynamics; moreover, the efficiency of ozonation device in the different operating conditions should be considered. This in-depth investigation will allow to evaluate the eventual up-scale feasibility of the system. Pilot studies on the field should be planned and executed to evaluate the operating conditions for agricultural irrigation, considering the specific features of the irrigation device in order to reduce clogging, forecasting proper filtration, or adding disinfectants (such as chlorine).

## Conclusions

The present study focused on a new circular economy approach for the water cycle with special attention to water/nutrients re-use by reduction of the current water consumption, contributing to the challenge of raw materials depletion.

A series of combined low-frequency ultrasound and ozonation tests were planned and executed to evaluate the potential reuse of a primary effluent from municipal wastewater treatment plant for civil, industrial, and agricultural applications. Ultrasound process as pre-treatment before ozonation demonstrated a good efficiency regarding COD and UV254 abatement. During the US-O_3_ combined treatment, the nutrient charge of the mixture, measured as total nitrogen and orthophosphate, was well-maintained: consequently, a high potential value for agricultural reuse of the treated primary effluent was highlighted, with possible significant saving of chemical fertilizers. Formaldehyde and MBAS parameters were reduced in the final effluent from the combined sonication-ozonation treatment, improving the high ozonation performances. Finally, primary effluent disinfection was more effective when a combined US-O_3_ treatment was applied, reaching maximum values of 5 logs on the Total Coliform parameter. It was concluded that low-frequency ultrasound pre-treatment, combined with ozonation, could be a useful process for primary effluent recovery for several purposes. Further studies are expected to be planned and executed to evaluate system scale-up feasibility and the detailed effects of the most meaningful process parameters on final effluent toxicity.
